# DNA Break Mapping Reveals Topoisomerase II Activity Genome-Wide

**DOI:** 10.3390/ijms150713111

**Published:** 2014-07-23

**Authors:** Laura Baranello, Fedor Kouzine, Damian Wojtowicz, Kairong Cui, Teresa M. Przytycka, Keji Zhao, David Levens

**Affiliations:** 1 Laboratory of Pathology, NCI/NIH, Bldg 10 Rm 2N106, 10 Center Drive, Bethesda, MD 20892, USA; E-Mails: baranellolf@mail.nih.gov (L.B.); levens@helix.nih.gov (D.L.); 2 Computational Biology Branch, NCBI/NLM/NIH, Bldg 38a, 8600 Rockville Pike, Bethesda, MD 20894, USA; E-Mails: wojtowda@mail.nih.gov (D.W.); przytyck@mail.nih.gov (T.M.P.); 3 Systems Biology Center, NHLBI/NIH, Bldg 10 Rm 7B06A, 10 Center Drive, Bethesda, MD 20892, USA; E-Mails: cuik@mail.nih.gov (K.C.); zhaok@mail.nih.gov (K.Z.)

**Keywords:** topoisomerases, DNA damage, transcription

## Abstract

Genomic DNA is under constant assault by endogenous and exogenous DNA damaging agents. DNA breakage can represent a major threat to genome integrity but can also be necessary for genome function. Here we present approaches to map DNA double-strand breaks (DSBs) and single-strand breaks (SSBs) at the genome-wide scale by two methods called DSB- and SSB-Seq, respectively. We tested these methods in human colon cancer cells and validated the results using the Topoisomerase II (Top2)-poisoning agent etoposide (ETO). Our results show that the combination of ETO treatment with break-mapping techniques is a powerful method to elaborate the pattern of Top2 enzymatic activity across the genome.

## 1. Introduction

Cells are constantly exposed to environmental and endogenous DNA damaging agents that compromise DNA integrity and threaten genomic stability. During physiological processes such as replication or transcription DNA aberrations arise as a consequence of base pair mismatches, R-loop formation and abortive activity of DNA breaking enzymes topoisomerases [1]. Moreover, genome integrity is continually challenged by physiological metabolites such as reactive oxygen species and environmental assaults such as radiation and ultraviolet light [2]. The most harmful lesions, DNA double-strand breaks, can trigger growth arrest or cell death [3,4] and are potent inducers of chromosomal rearrangements such as deletions, translocations and amplifications [5]. Proliferation of the damaged cells results in variety of diseases, including cancer and premature aging. Single-strand breaks (SSBs) can impair the progression of the transcriptional apparatus and the replication machinery can collide with SSBs leading to the formation of lethal double-strand breaks (DSBs) [6]. Nevertheless, emerging evidence suggests that in some instances DNA breakage can support transcriptional activation, as shown for the *Hsp70* gene in Drosophila or the pS2 promoter in human cells [7,8], where Topoisomerase II (Top2)-mediated breakage alters the nucleosome structure of the promoter and triggers transcription. This evokes the unexpected concept of DNA cleavage as a regulatory element in genome functioning.

Most of the techniques developed to study DNA breaks are based on their indirect detection. Through the localization of proteins that bind the breaks such as the phosphorylated histone variant γH2AX [9] or the replication protein A (RPA), or by detecting the single-stranded DNA that transiently accumulates at DSB sites [10], the biology of DNA breaks has been investigated. However, despite past work providing comprehensive information on the pathways involved in detecting and repairing DNA breakage, the genomic landscape of DSBs and SSBs remains poorly understood. Recently a method based on direct *in situ* labeling of DSBs has provided insight into the “breakome” in different conditions, revealing that even in the absence of exogenous treatment human cells carry a substantial number of breaks [11]. However, the method was not implemented to identify SSBs and place the breakage directly within the genomic sequence context [2].

The topological state of the DNA is regulated by enzymes known as topoisomerases which are required for genome functioning [12]. Topoisomerases modulate DNA topology by generating transient breaks in the double helix. There are two major classes of Topoisomerases, type I (Top1) and type II (Top2), that are distinguished by the number of DNA strands that they cleave and the mechanism by which they alter the topology of DNA. In particular, Top2 generates transient DSBs in DNA and consequently has the capacity to damage the genome during the enzymatic reaction until the breaks are re-ligated. Treatment with drugs that poison Top2 before ligation of DNA is among the most successful chemotherapeutic approaches to kill cancer cells [13]. Though recent studies have expanded the biological contexts for Top2 function, a technique detecting its enzymatic activity through the genomic identification of DNA cleavage was lacking [14].

Here we present two simple approaches to map DSBs and SSBs across the genome. These are based on the direct labeling of breaks with two independent strategies: 3'-end tailing of DSBs with biotin-modified nucleotides catalyzed by terminal deoxynucleotidyl transferase (TdT), and nick translation of the SSBs extremity with DNA polymerase I in the presence of digoxigenin-modified nucleotides. We tested each technique on HCT116 cells treated with the DNA damaging agent etoposide (ETO), an anti-cancer drug that inhibits the Top2 catalytic cycle. Overlaying the distribution of breaks along genes and comparing with the level of gene expression showed that Top2-associated breaks preferentially localize at promoters of high and medium expressed genes. These results expand our understanding of Top2 function during transcription, providing details about its activity genome-wide.

## 2. Results and Discussion

### 2.1. Overview of Single-Strand Break (SSB)-Seq

**Figure 1:**
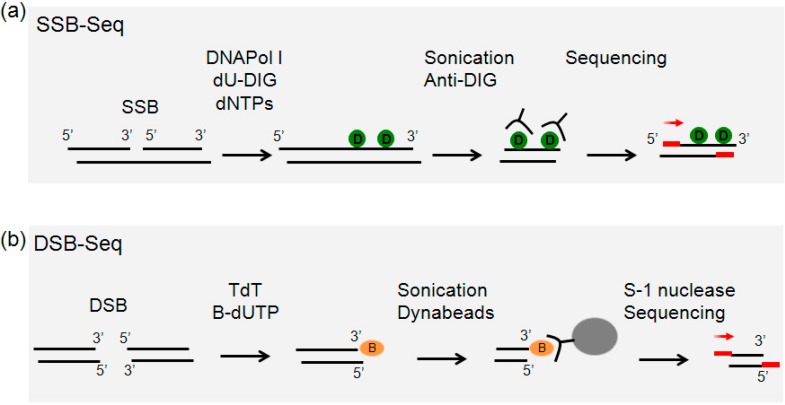
(**a**) Single-strand breaks (SSBs) are labelled during nick translation using nucleotides covalently linked to digoxigenin (green circle). Genomic DNA is purified, sonicated and immunoprecipitated with anti-digoxigenin antibody (anti-DIG). Precipitated DNA is sequenced; (**b**) 3' tails of double-strand breaks (DSBs) are ligated to biotinylated nucleotides (orange circle) and after sonication the labelled fragments are captured on streptavidin beads (gray circle). Tails are removed from released fragments and DNA is sequenced. Red bars represent the Illumina adaptors (see Experimental Section for details). Red arrows represent the direction of sequencing.

High molecular weight genomic DNA was isolated from cells lysed in the presence of SDS and proteinase K extracted twice with phenol-chloroform, and precipitated with ethanol in presence of ammonium acetate. Samples were subjected to nick-translation by DNA polymerase I in the presence of deoxynucleotide triphosphates including digoxigenin labeled dUTP. To restrict labeling to a small patch of DNA in order to increase the resolution of mapping, dideoxynucleotides were included in the reaction to inhibit excessive chain elongation by DNA polymerase I. As a control for the labeling, samples were also nick-translated without digoxigenin-labled nucleotides. DNA was sheared by sonication and labeled fragments were immunoprecipitated with anti-digoxigenin antibody, purified and subjected to Illumina library preparation and sequencing (Figure 1a).

### 2.2. Overview of Double-Strand Break (DSB)-Seq

After isolating high molecular weight genomic DNA as above, the double-stranded DNA ends were 3'-end tailed with TdT in the presence of biotinylated nucleotides. Finally, samples were sonicated, and biotinylated DNA was streptavidin-selected. In parallel, as a negative-control for labeling and selection, 3'-tailing was performed in the absence of biotinylated nucleotides. To remove the biotinylated-tails, samples were treated with S-1 nuclease; the resulting DNA was purified and subjected to Illumina library preparation and sequencing (Figure 1b).

### 2.3. Etoposide Induces SSBs

**Figure 2:**
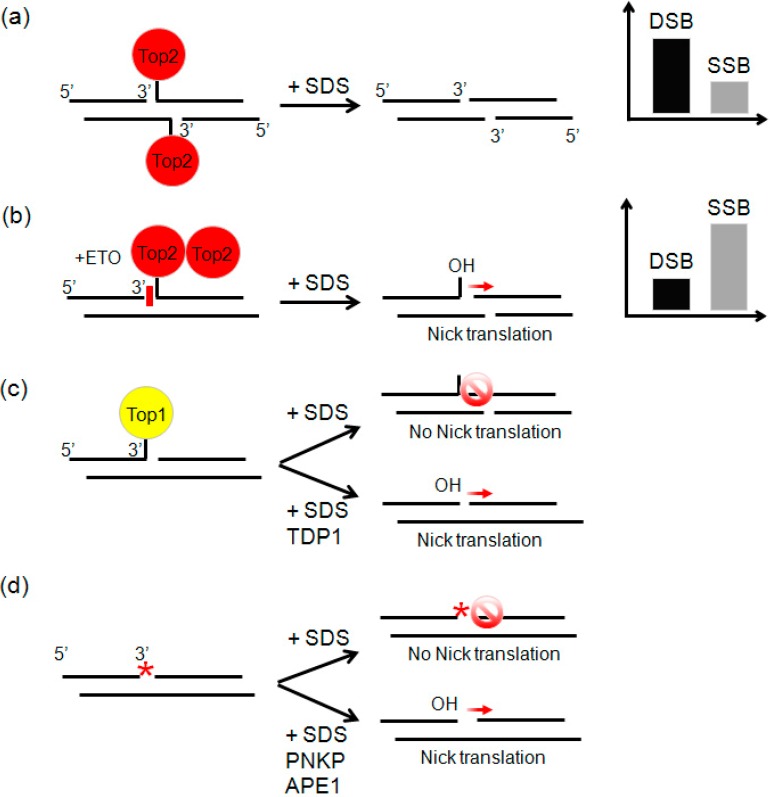
DSBs (**a**) and SSBs (**b**–**d**) generated in presence of Top2 (**a**); ETO (**b**); Top1 (**c**); and DNA-damaging agents that modify the DNA termini (**d**). Red arrow represents successful nick translation. Stop sign represents unsuccessful nick translation. Nick translation by DNA polymerase I necessitates a 3'-OH, which is not reconstituted in case of Top1 cleavage or when the DNA termini is damaged (shown by asterisk). In these cases the principal enzymes involved in processing and repair of the ends are listed below the black arrow. TDP1, tyrosyl-DNA phosphodiesterase 1, PNKP, polynucleotide kinase 3'-phosphatase, APE1, AP endonuclease I [17]

To demonstrate the specificity and sensitivity of SSBs and DSBs capture, we performed a pilot experiment on HCT116 cells briefly treated with ETO. The drug stabilizes the covalent Top2-cleaved DNA complex that is a transient intermediate in the catalytic cycle of the enzyme. Because Top2 is a homodimer with each monomer cleaving a single DNA strand, denaturation of topoisomerase in the complex with DNA by SDS (Figure 2a) could result in a permanent DSB [15]. Since the drug inhibits each Top2 subunit independently, contrary to conventional expectation, ETO treatment generates mostly SSBs, to the detriment of DSBs (Figure 2b). SSBs occur when only one Top2 monomer is inhibited, leaving the other subunit free to rejoin the DNA ends [16]. Top2 performs DNA cleavage via tyrosyl-active site residues, establishing a covalent phosphotyrosyl bond linking the enzyme to the 5'-terminus of the DNA. It also generates a 3'-hydroxyl moiety on the opposite terminus of the cleaved strand, which is a proper substrate for nick-translation. Consequently, SSB-Seq in parallel with ETO treatment is highly specific for Top2 activity. This procedure does not detect SSBs resulting from Topoisomerase I (Top1) activity or SSBs bearing damaged DNA termini unless processed by DNA-repairing enzymes (Figure 2d) [17]. Within the Top1–DNA cleavable complex, the enzyme is covalently bound with the phosphate to the 3' end of DNA (Figure 2c). To detect these SSBs it would be necessary to reconstitute a free 3'-OH terminus to serve as a substrate for DNA polymerase I. Therefore, even if Top1 activity were indirectly altered in cells due to Top2 inhibition, the SSBs detected in our experiments, reflect only catalytically engaged Top2.

**Figure 3:**
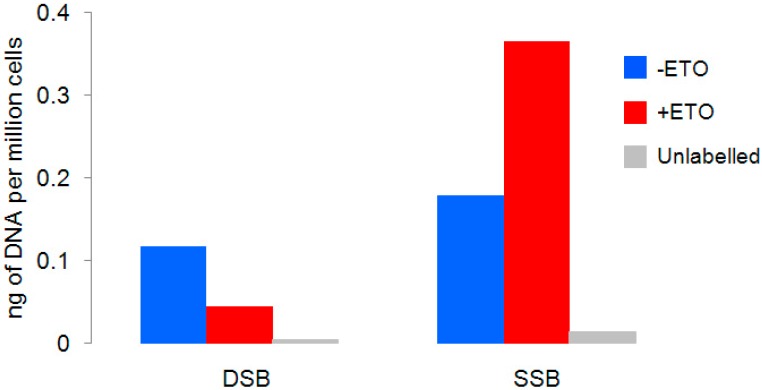
DNA quantification of recovered DNAs after SSB- and DSB-Seq in the presence (+ETO) and in the absence (−ETO) of 20 μM of etoposide. The “unlabelled” samples were not incubated with digoxigenin- or biotinylated-nucleotides. The recovered DNA is normalized to the number of cells.

To validate our experimental approach, we briefly exposed cells to a low dose of ETO, labelled SSBs and DSBs and quantified the DNA recovered after antibody/streptavidin selection. As expected, cells exposed to the drug accumulate more SSBs, compared to the untreated sample (Figure 3). On the contrary, the amount of DBSs was lower than the untreated control.

### 2.4. Promoters Are Hot Spots of DNA Breaks

**Figure 4:**
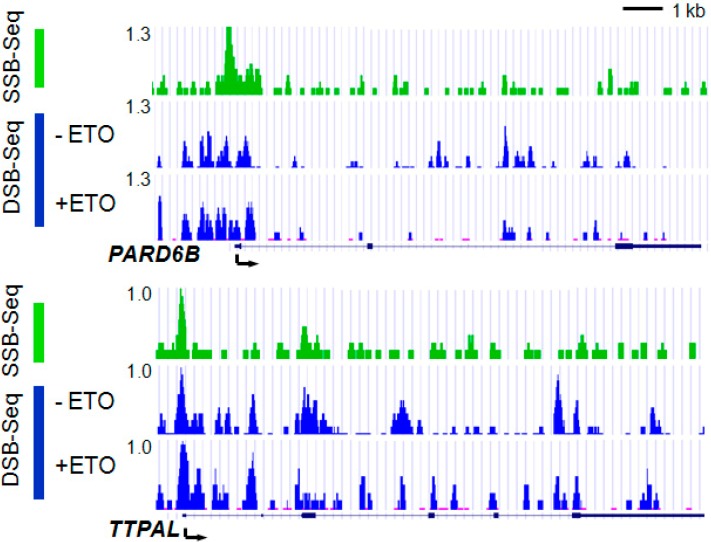
SSB (green) and DSB (blue) profiles at two representative genes (*PARD6B* and *TTPAL*) randomly selected. The data are displayed as custom tracks on the UCSC genome browser (University of California, Santa Cruz, CA, USA). The positions of the genes are indicated below the panel. The *y*-axis shows the number of tags per million reads and the *x*-axis shows the chromosome coordinates in the genome. Cells were treated with ETO (20 μM for 5 min). Due to the smaller amount of the double-stranded breaks in the genome the DSB profile looks noisier than the SSB profile. Exons are depicted as boxes and introns as lines.

After validation, we next applied the SSB- and DSB-Seq to map Top2 activity genome-wide. Top2 is functional in a wide range of biological contexts such as DNA replication, chromosome segregation and transcription [18]. The twin supercoiling model [19] provides an important rationale for the Top2-requirement during transcriptional elongation where transcribing RNA polymerase twists the DNA through its active site, driving torsional stress ahead and behind it. However, how and where Top2 works during transcription remains poorly investigated. The relationship between transcription and Top2-associated breaks was revealed by deep-sequencing the DNA from SSB-Seq and DSB-Seq and showed that DNA breaks displayed a striking preference for the 5'-end of the genes (Figure 4). As expected, we observed a similar pattern for DSBs and SSBs, indicating that during genomic DNA preparation the main pool of DNA double-stranded breaks results from the enzymatic activity of Top2.

**Figure 5:**
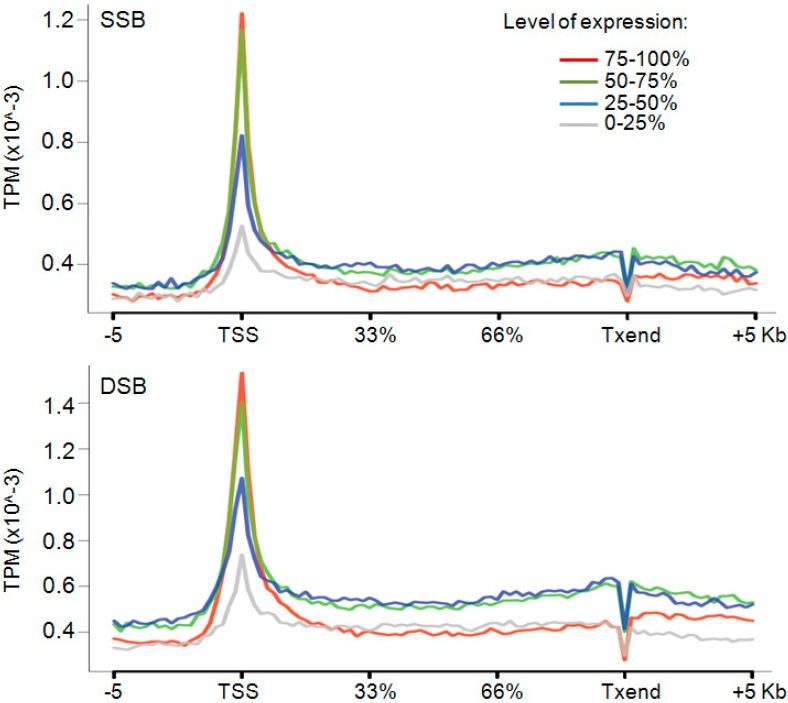
SSBs (top panel) and DSBs (bottom panels) density across genes ranked in 4 percentiles (0%–25%–50%–75%–100%). Data represented as sequence tags per million (TPM).

As postulated in the twin supercoiled domain model, the increase in gene expression should increase the level of transcription-generated torsional stress and require more Top2. Therefore we computed the break-density at genes with high, medium and low output, as measured by microarray experiments [20]. The analysis revealed that Top2-breaks were most enriched at transcriptional start sites (TSS) compared to the rest of the gene. Notably, genes with elevated expression exhibited higher frequency of Top2-breakage than did lower expressed or silent genes (Figure 5). These observations complement and extend a recent work showing for a small set of the genes that Top2 preferentially binds promoters of the highly expressed genes [21]. Overall these finding suggest that Top2 exerts its enzymatic activity mainly at TSS in a manner dependent on the level of promoter output. These results also indicate that the sites of breakage in standard DNA preparations may reflect Top2 halted in mid-catalytic cycle at the time of cell lysis.

DNA breakage is intrinsically linked to the genome biology. Breaks can be hazardous for genome stability but also a means to alter the arrangement, conformation and topology of chromatin [14]. Our methods provide a useful tool to map SSBs and DSBs in different cells and physiological or pathological conditions, on a genome-wide scale. Although past research, mainly based on chromatin immunoprecipitation (ChIP) approach, has provided some information about Top2 localization [21], these studies cannot discriminate binding from activity. Previous works shows that Top2 activity is favored by the crossing of DNA segments [22], which likely occurs at promoters where plectonemes form in negatively supercoiled DNA unbuffered by chromatin rearrangement [23]. Accordingly, we showed that the majority of Top2-associated breaks occur at promoters where high negative torsional stress accumulates in the wake of RNA polymerase movement [21].

## 3. Experimental Section

### 3.1. Cells and Reagents

Human colon cancer cells HCT116 were grown in DMEM supplemented with 10% heat-inactivated FCS. Etoposide (Sigma, St. Louis, MO, USA) treatment was performed on exponentially growing cells at 37 °C for 20 min with a final concentration of 20 μM.

### 3.2. Purification of High Molecular Weight DNA

To prepare high molecular weight DNA, 8 × 10^7^ cells were washed twice with ice-cold PBS and lysed with 10 mL of lysis buffer (10 mM Tris–HCl pH 8.0, 100 mM EDTA pH 8.0, 0.5% SDS). Lysates were collected, digested overnight with 200 μg/mL of proteinase K (Roche, Indianapolis, IN, USA) at 52 °C and DNA was extracted twice with phenol, once with phenol chloroform, and then ethanol precipitated in the presence of 2 M ammonium acetate (Sigma). The sample was then treated with 5 μg of pancreatic RNase (Roche) for 1 h at 37 °C, adjusted to 0.5% SDS and incubated for 1 h at 55 °C with proteinase K (200 μg/mL). DNA was extracted twice with phenol and precipitated with ethanol in the presence of 2 M ammonium acetate. Finally, the pellet was resuspended in 1 mL of TE (10 mM Tris–HCl, 1 mM EDTA pH 8.0) and incubated at room temperature for 12 h with gentle rotation. DNA size was determined by gel electrophoresis, and its concentration was measured with a NanoDrop ND-1000 spectrophotometer (Thermo Scientific, Rochester, NY, USA).

### 3.3. SSB-Seq

SSBs were labeled by nick translation. 500 μg of DNA was incubated for 40 s at 16 °C with a mixture of 200 μM of dATP, dGTP, dCTP and 20 μM of digoxigenin-11-dUTP (Roche), 117 μM of ddATP, ddGTP, ddCTP (Roche) and 1000 units of *Escherichia coli* DNA Polymerase I (New England Biolabs, Ipswich, MA, USA). As a control for labeling, 500 μg of DNA was incubated with the same reagents except digoxigenin-11-dUTP was substituted by 20 μM of dTTP. The reactions were stopped with 50 μM EDTA and DNA purified with phenol, precipitated twice in the presence of ethanol and 2.5 M ammonium acetate, and DNA was sheared by sonication to an average fragment size of 200–400 bp. Sonication was performed with an ultrasonic sonicator Bioruptor (Diagenode, Denville, NJ, USA) by pulsing 30 times for 30 s at medium power and incubating on ice for 30 s between each pulse. The samples were incubated at 4 °C overnight with 10 μg of Anti-digoxigenin antibody (Roche) with gentle rotation. Immunocomplexes were recovered by addition of 60 μL of Protein G-Sepharose beads (Roche) and incubated for 4 h at 4 °C. The beads were washed once with PBS, three times with NP-40 buffer (20 mM Tris–HCl pH 8.0, 137 mM NaCl, 10% Glycerol, 1% NP-40, 2 mM EDTA pH 8.0); twice with TE (10 mM Tris–HCl, 1 mM EDTA pH 8.0); and finally resuspended in 200 μL of TE. Each wash was performed for 10 min by gentle agitation followed by 4 min of centrifugation at 1500× *g*. The pellets were adjusted to 0.5% SDS and digested with proteinase K (200 μg/mL) at 65 °C overnight. DNA was purified using QIAquick PCR Purification Kit Protocol (QIAGEN, Valencia, CA, USA) according to the manufacturer’s instructions and quantified. 

### 3.4. DSB-Seq

This method was modified from [24]. To label DSBs 500 μg of DNA was biotinylated by 3'-end tailing reaction in 3 mL of TdT buffer (Roche) with 24,000 U TdT (Roche), 0.5 mM dCTP, and 0.02 mM Biotin-16-dUTP (Roche) at 37 °C for 30 min. Reactions were stopped by adding EDTA to a 20 mM final concentration. As a control of labeling, 500 μg of DNA was incubated with the same reagents substituting TTP for Biotin-16-dUTP. To remove unincorporated biotin, samples were extracted with phenol chloroform, precipitated twice with 2 M ammonium acetate and ethanol and dissolved in 200 mL of TE buffer. Biotinylated DNA was sonicated, as above, to generate 200–400 bp DNA fragments. Then, biotinylated fragments were captured with streptavidin-coated beads by using Dynabeads kilobaseBINDER Kit (Invitrogen Dynal, Oslo, Norway) according to the manufacturer’s protocol. After 4 washes at 50° and 4 washes at room temperature with 10 mM Tris–HCl pH 7.5, 1.0 mM EDTA, and 2.0 M NaCl, biotin-streptavidin complexes were disrupted by incubating them in 10 mM Tris–HCl pH 7.5, 1 mM EDTA, 1 M NaCl, and 2 M β-mercaptoethanol at 75 °C for 4 h. Free DNA fragments were purified with a QIAquick PCR Purification Kit (QIAGEN). To remove biotinylated tails from DNA, samples were incubated with 30 U of S1 nuclease (Fermentas, Clen Burnie, MD, USA) in 110 μL of recommended buffer for 30 min at 37 °C. DNA was purified with a QIAquick PCR Purification Kit (QIAGEN).

### 3.5. Template Preparation for Sequencing Analysis

The Epicentre DNA END-Repair kit (Epicentre Biotechnologies, Madison, WI, USA) was used to generate blunt-ended DNA. DNA was incubated for 45 min at room temperature with a mixture of End repair buffer (33 mM Tris–acetate pH 7, 66 mM potassium acetate, 10 mM magnesium acetate, 0.5 mM DTT), 0.25 mM of each dNTPs, 1 mM ATP, and 1 μL End-Repair Enzyme mix (T4 DNA polymerase + T4 PNK). After purification, the blunt-ended DNA was treated with 15 units of Klenow (exo-) for 30 min at 37 °C in the presence of 0.2 mM dATP to generate a protruding 3'-A base used for adaptor ligation. Illumina adapter was ligated to the end of DNA fragments by incubating with 0.1 μL Adaptor oligo mix and 1000 units of T4 DNA ligase at room temperature for 30 min. After one step of DNA purification using QIAquick PCR Purification Kit (QIAGEN) the DNA was eluted. A size selection of the adapter ligated DNA was performed through 2% E-Gel (Invitrogen, Carlsbad, CA, USA) electrophoresis. The gel slice, around the 200–400 bp region was excised, then DNA was extracted using the MinElute gel extraction kit (QIAGEN) in a final volume of 12 μL elution buffer. The DNA was then amplified for 18 cycles using Illumina primers (Fw: 5'-ACACTCTTTCCCTACACGACGC-3'/Rv: 5'-CAAGCAGAAGACGGCATACGAGC-3') according to the following protocol: 98 °C for 30 s; 65 °C for 30 s; 72 °C for 30 s. The PCR product was obtained by excising 220–500 bps DNA from a 2.5% agarose gel and purifying it through a Qiagen gel extraction kit (QIAGEN). The purified DNA was used directly for cluster generation and sequencing analysis using the Illumina Genome Analyzer (Illumina, San Diego, USA) following manufacturer protocols.

### 3.6. Data Analysis of Microarrays

The oligo microarray data (Affymetrix Human Genome U133 Plus 2.0 Array) of total RNA from HCT116 cells (four replicates) were retrieved from the NCBI Gene Expression Omnibus (National Center for Biotechnology Information, Bethesda, MD, USA) under the GEO accession number GSE7161. CEL files were analyzed using R environment (Free Software Foundation, Boston, MA, USA) with Bioconductor package (Fred Hutchinson Cancer Research Center, Seattle, WA, USA). We used a GeneChip robust multi-array analysis, *gcrma,* method for background correction and normalization of the raw probe measurements to attain expression values. The maximum expression of Affymetrix U133 Plus 2.0 identifiers (Affymetrix, Santa Clara, CA, USA) mapped to human genes annotated in Ensembl 60 [25] was chosen as the gene expression value. Genes were split into quartile groups based on their expression values.

### 3.7. Processing of Sequencing Data

Sequencing data were preprocessed using the Illumina Analysis Pipeline (image analysis and base calling, Illumina, San Diego, CA, USA). The short reads of length 36 bp were aligned to the human genome (hg19) using Bowtie 2 program (version 2.2.2) [26]. To minimize potential PCR bias redundant reads were removed. The profiles of average distribution of sequencing reads along normalized protein-coding gene bodies were generated using ngs.plot (version 2.0.8) [27].

## 4. Conclusions

The methodology described in this communication provides a fast and simple approach for the study of genome-wide Top2 enzymatic activity and function in various cells and experimental conditions. Using etoposide treatment we map the activity of both Top2 isoforms α and β; combining SSBs- and DSBs-Seq with more specific inhibitors will allow the development of genome-scale maps revealing finer structures such as the distributions of these different isoforms. For instance doxorubicin, another active anticancer drug, preferentially targets cellular Top2α over Top2β [28,29,30]. Finally, these break-mapping techniques could prove useful in genotoxic drug screens to map the breakome resulting from treatment with anticancer drugs, many of which are topoisomerase inhibitors.
